# Inhibition of sphingosine 1-phosphate receptor 3 ameliorates bleomycin-induced pulmonary fibrosis by suppressing macrophage M2 polarization

**DOI:** 10.1016/j.gendis.2024.101244

**Published:** 2024-02-15

**Authors:** Huijun Qiu, Jiang Liu, Jingyi You, Ou Zhou, Chang Hao, Yi Shu, Deyu Ma, Wenjing Zou, Linghuan Zhang, Enmei Liu, Zhengxiu Luo, Luo Ren, Gang Geng, Lin Zou, Danyi Peng, Zhou Fu

**Affiliations:** a Department of Respiratory Medicine, Children's Hospital of Chongqing Medical University, National Clinical Research Center for Child Health and Disorders, Ministry of Education Key Laboratory of Child Development and Disorders, Chongqing Key Laboratory of Pediatrics, Chongqing 400014, China; b Department of Neonatology, Children's Hospital of Chongqing Medical University, Chongqing 400014, China; c Department of Otolaryngology, Children's Hospital of Chongqing Medical University, Chongqing 400014, China; d Center of Clinical Molecular Medicine, Children's Hospital of Chongqing Medical University, Chongqing 400014, China; e Clinical Research Unit, Institute of Pediatric Infection, Immunity and Critical Care Medicine, Children's Hospital of Shanghai Jiaotong University Medical School, Shanghai 200062, China

**Keywords:** Bleomycin, IL-4, M2 polarization, Macrophage, Pulmonary fibrosis, S1pr3

## Abstract

Pulmonary fibrosis is a devastating lung disease without effective treatment options. Sphingosine-1-phosphate receptor 3 (S1pr3), a receptor for the lipid signaling molecule sphingosine-1-phosphate, has been shown to mediate the development of pulmonary fibrosis, although the underlying mechanism is not fully understood. Here, we found increased expression of S1pr3 in the lung during the process of bleomycin-induced pulmonary fibrosis in mice and specific overexpression of S1pr3 in the infiltrated M2 macrophages. We constructed *LysM-Cre*^*+*^*/S1pr3*^*flox/flox*^ mice, in which *S1pr3* was conditionally depleted in myeloid cells, and this depletion protected mice from bleomycin-induced lung injury and fibrosis, with reduced M2 macrophage accumulation in the lung. Increased S1pr3 expression was found in bone marrow-derived macrophages after alternatively activated by IL4 *ex vivo*, while loss of *S1pr3* attenuated IL-4-induced M2 polarization in bone marrow-derived macrophages by repressing the PI3K/Akt-Stat3 signaling pathway. Moreover, the S1pr3 inhibitors CAY10444 and TY52156 exerted protective effects on pulmonary fibrosis in mice. Taken together, our research showed that inhibition of S1pr3 ameliorates bleomycin-induced pulmonary fibrosis by reducing macrophage M2 polarization via the PI3K/Akt-Stat3 signaling pathway, indicating that S1pr3 may be a potential target for pulmonary fibrosis treatment.

## Introduction

Pulmonary fibrosis (PF) is a devastating lung disease characterized by excessive accumulation of extracellular matrix, which results in distortion of the lung architecture, decline of lung function, and ultimately respiratory failure and death,^1^ Although the newly launched drugs pirfenidone and nintedanib both have certain therapeutic effects on the deterioration of lung function and disease progression in the patients with idiopathic PF, there is no effective therapy to reduce the mortality rate of PF.^2^ Meanwhile, the incidence and mortality rates of PF have increased worldwide over time,^3^ causing a considerable economic and health burden. The molecular mechanisms of PF remain poorly understood despite years of research on its pathogenesis. The formation of fibrosis is an abnormal reparative process, for which an early inflammatory response is regarded as an initiating trigger.^4^^,^^5^ Macrophages are the most abundant inflammatory cell type in the lung under normal conditions^6^ and account for more than 90% of cells in bronchoalveolar lavage fluid from fibrotic lungs.^7^ Macrophages are highly plastic and diverse and can be polarized to the classically activated phenotype (M1) or alternatively activated phenotype (M2) according to the microenvironment.^8^^,^^9^ The critical role of M2 macrophages in the development of fibrosis is well established; M2 macrophages can exert pro-fibrotic effects by secreting TGF-β and multiple growth factors,^10^, ^11^, ^12^, ^13^ which in turn stimulate the differentiation of fibroblasts into myofibroblasts to facilitate the synthesis of extracellular matrix.^14^ Therefore, controlling the number and phenotype of macrophages may be a viable therapeutic strategy, in which identifying the underlying mechanisms of macrophage M2 polarization is a critical step.

Sphingosine-1-phosphate (S1P) is a bioactive sphingolipid that binds to five related G protein-coupled receptors, named S1P receptors^15^ (S1PR1–5), to regulate diverse cellular processes, including cell growth, survival, and differentiation, and cytokine and chemokine production.^16^ The S1PR1, S1PR2, and S1PR3 subtypes are expressed essentially everywhere in the body, whereas the expression of S1PR4 and S1PR5 is much more restricted.^17^ In the past decade, S1P signaling has been recognized as a vital regulator of fibrotic diseases. Patients with idiopathic PF have increased S1P levels in serum and bronchoalveolar lavage fluid,^18^ which leads to the activation of S1PRs in interstitial cells and infiltrating immune cells to promote fibrotic changes.

Among S1PRs, S1PR3 mediates the development of PF, and *S1pr3-*deficient mice exhibit attenuated inflammation and lung fibrosis after bleomycin (BLM) challenge through decreased connective tissue growth factor.^19^ In mice, *S1pr3* knockdown in the lung has been shown to attenuate radiation-induced PF via miR-495-3p.^20^ S1pr3 expression levels have been reported to be increased in macrophages after IL-4 stimulation *in vitro*.^21^ However, little research has focused on the effect of S1pr3 on macrophages in the context of PF, which is pivotal for fibrosis formation.

In our study, we observed elevated S1pr3 expression levels following BLM challenge, with peak expression noted at the early inflammation stage. Interestingly, M2 macrophages were also found to accumulate in fibrotic lungs and express S1pr3. Moreover, inhibition of S1pr3 by knockout or inhibitors ameliorated BLM-induced PF in mice accompanied by a marked decrease in M2 macrophage infiltration into the lung. *Ex vivo* experiments illustrated that *S1pr3* deficiency attenuates macrophage M2 polarization by repressing the PI3K/Akt-Stat3 signaling pathway.

## Materials and methods

### Animals

In this study, PF was induced in 8-week-old male C57BL/6 mice. Mice were fed adaptively for at least 3 days before the experiments. Mice were anesthetized with 1% pentobarbital sodium and then intratracheally injected with 2 mg/kg BLM (Hanhui Pharmaceuticals Co., Ltd., China); the day that BLM was administered was considered day 0 (D0). Normal control mice were intratracheally injected with an equal amount of 0.9% saline solution. Mice were sacrificed on days 3 (D3), 7 (D7), and 14 (D14) to detect S1pr3 expression. For the intervention experiments, the S1pr3 inhibitors CAY10444 (10 mg/kg) or TY52156 (10 mg/kg) were injected intraperitoneally^22^ from day 1 after BLM administration and were given every other day. Dimethyl sulfoxide was administered intraperitoneally as a solvent control. Mice were sacrificed on day 14.

*LysM-Cre* mice (strain No. T003822) and *S1pr3-flox* mice (strain No. T006388) were purchased from GemPharmatech (Nanjing, China). *LysM-Cre*^*+*^*/S1pr3*^*flox/flox*^ (defined as *S1pr3-CKO*) mice were generated by crossing *LysM*-*Cre* mice with *S1pr3*^*flox/flox*^ mice to specifically deplete *S1pr3* in myeloid cells, while their littermates *LysM-Cre*^*−*^*/S1pr3*^*flox/flox*^ (defined as *S1pr3-C*) mice were used as controls. *S1pr3-CKO* and *S1pr3-C* mice were intratracheally injected with 2 mg/kg BLM to induce PF and sacrificed on day 14. All animals were maintained on 12-h dark/light cycles and were provided with water and a standard rodent diet *ad libitum*. The animal use protocol was reviewed and approved by the Ethics Committee of the Children's Hospital of Chongqing Medical University (No. CHCMU-IACUC20211222003).

### Histopathologic analyses

The left lungs were fixed in a 4% paraformaldehyde solution, dehydrated in a graded ethanol series, embedded in paraffin, and sectioned at a thickness of 4 μm. Sections were stained with hematoxylin–eosin (HE) or Masson's trichrome. Whole slides were scanned using a slide scanning system (TEKSQRAY, Shenzhen, China). Quantitative assessments of fibrotic changes were evaluated using the Ashcroft score with a modified scale.^23^

### Hydroxyproline measurement

The measurement of hydroxyproline was conducted using a hydroxyproline measurement kit (NanJing JianCheng Bioengineering Institute) according to the manufacturer's instructions. Approximately 40 mg (wet weight) of lung tissue was collected, 1 mL of alkaline hydrolysate was added, and the mixture was boiled at 100 °C for 20 min. The reagent provided was used to adjust the pH to 6.0–6.8. Approximately 3–4 mL of supernatant was collected for measurement after sorption onto active carbon. The hydrolysate was centrifuged at 3500 rpm for 10 min. Finally, 1 mL of the supernatant was taken for measurement according to the manufacturer's instructions.

### Cell culture and treatment

Primary bone marrow-derived macrophages (BMDMs) were obtained by flushing mouse femurs and tibias and cultured in RPMI-1640 medium with 10% fetal bovine serum, 1% solution of penicillin and streptomycin, and 20 ng/mL macrophage colony-stimulating factor. The medium was changed every 2 days. All cells were cultured at 37 °C in a humid atmosphere containing 5% CO_2_. After 7 days, the differentiated macrophages were starved overnight and then treated with 20 ng/mL IL-4 for the specified durations.

### Western blot

Lung tissue and cell proteins were extracted with a total protein extraction kit (KeyGenBiotech, China) according to the manufacturer's instructions. The extracted protein samples were separated via sodium dodecyl sulfate-polyacrylamide gel electrophoresis and transferred to polyvinylidene fluoride membranes. Subsequently, the membranes were blocked with 5% skim milk for at least 1 h at room temperature, before incubation with the following antibodies: anti-S1PR3 (1:800, Genetex, China), anti-fibronectin (1:1000, Proteintech, China), anti-collagen I (1:1000, Proteintech, China), anti-arginase1 (1:1000, Proteintech, China), anti-iNOS (1:1000, Abcam, USA), anti-PI3K (1:1000, CST, USA), anti-p-PI3K (1:1000, Bioss, China), anti-AKT (1:1000, CST, USA), anti-p-AKT (1:1000, CST, USA), anti-STAT3 (1:1000, Proteintech, China), anti-p-STAT3 (1:1000, Proteintech, China), anti-GAPDH (1:5000, Proteintech, China), and anti-α-tubulin (1:5000, Proteintech, China) at 4 °C overnight. The membranes were treated for 1 h with horseradish peroxidase-conjugated secondary antibody at room temperature. After the membranes were washed with Tris-buffered saline containing 0.05% Tween 20 detergent, the bands were detected using an enhanced chemiluminescence reagent. Quantitative analyses were conducted using Image J.

### Immunohistochemistry

Lung tissue sections were deparaffinized and boiled at 95 °C for 30 min in 0.05% citrate buffer (pH 6.0) for antigen retrieval. The immunohistochemistry analysis kit (ZSGB-BIO, Beijing, China) was applied according to the manufacturer's protocol. Briefly, tissue sections were immersed in endogenous peroxidase blocking solution for 10 min, blocked for 1 h using 10% normal goat serum, and then incubated overnight with anti-arginase1 (1:200, Proteintech, China) at 4 °C. Sections were immersed for 20 min in reaction enhancer solution and then incubated with horseradish peroxidase-conjugated secondary antibody for 20 min. DAB chromogen kit was used to visualize the positively stained cells, and hematoxylin was used to counterstain the nuclei. Stained slides were digitalized on a slide scanning system and then analyzed with ImageJ.

### Immunofluorescence staining

To confirm the purity of BMDMs, primary bone marrow cells were seeded on a 24-well plate containing sterile round glass coverslips at a density of 5 × 10^5^ cells/well. After 7 days of differentiation, adherent cells were washed with precooled phosphate-buffered saline solution and fixed with cold methanol at room temperature for 10 min. After washing, the cells were blocked with 10% normal goat serum for 1 h and then incubated with FITC-labeled anti-F4/80 (1:100, eBioscience, USA) in the dark for 1 h. Next, 4′,6-diamidino-2-phenylindole was used to visualize the nuclei. Fluorescence was observed using a Leica laser confocal microscope (C2+ system, Nikon, Japan). For the co-immunostaining experiment, lung tissue sections were deparaffinized and boiled at 95 °C for 30 min in 0.05% citrate buffer (pH 6.0) for antigen retrieval. Subsequently, the sections were blocked with 10% normal goat serum. For the co-immunostaining of CD115 and S1pr3, the lung tissue sections were stained with a mixture of mouse anti-CD115 (1:50, Novus, USA) and rabbit anti-S1PR3 (1:50, Zenbio, China) at 4 °C overnight, followed by exposure to a mixture of goat anti-mouse IgG FITC secondary antibody (1:200, Proteintech, China) and goat anti-rabbit IgG Cy3 secondary antibody (1:200, Proteintech, China) in the dark for 1 h. When it came to the co-immunostaining of Ly-6G and S1pr3, the lung tissue sections were stained with rabbit anti-S1PR3 (1:50, Zenbio, China) at 4 °C overnight, followed by exposure to a mixture of APC-labeled anti-Ly-6G (1:100, eBioscience, USA) and goat anti-rabbit IgG FITC secondary antibody (1:200, Proteintech, China) in the dark for 1 h. 4′,6-diamidino-2-phenylindole was used to visualize the nuclei. Fluorescence was observed using a Leica laser confocal microscope (C2+ system, Nikon, Japan).

### Flow cytometry analysis

BMDMs were stimulated with 20 ng/mL IL-4 for 24 h. The cells were then stained with a mixture of anti-F4/80-FITC (1:100, eBioscience, USA), CD11b-PerCP/Cy5.5 (1:100, eBioscience, USA), CD11c-V450 (1:100, eBioscience, USA), and CD206-APC (1:100, Invitrogen, USA) antibodies. After washing, the cells were analyzed with a FACS Canto II (BD Biosciences, San Jose, CA, USA). The gating strategy was as described previously.^24^ All data were analyzed using FlowJo software (Tree Star, Inc.).

### Quantitative reverse transcription-PCR

Total RNA from lung tissues and cells was extracted with TRIzol reagent (TaKaRa, Tokyo, Japan), cDNA was synthesized with the cDNA reverse transcription kit (NEB, USA), and quantitative reverse transcription PCR (RT-PCR) was performed using SYBR Premix Ex Taq (CWbio, China). Gene expression levels were calculated using the 2^−ΔΔCT^ method. GAPDH was used as an endogenous control.

### Statistical analysis

All data were presented as mean ± standard error of the mean. Statistical analysis was performed using GraphPad Prism 8.0 software (GraphPad, San Diego, CA, USA). The two experimental groups were compared using Student's *t*-test for paired data or Student's *t*-test with Welch's correction for unpaired data. For comparisons of more than two groups, a one-way analysis of variance followed by Tukey's post hoc test was conducted. *P*-values <0.05 were considered statistically significant.

## Results

### S1pr3 is up-regulated in the lungs during the development of BLM-induced PF in mice

**Figure 1:**
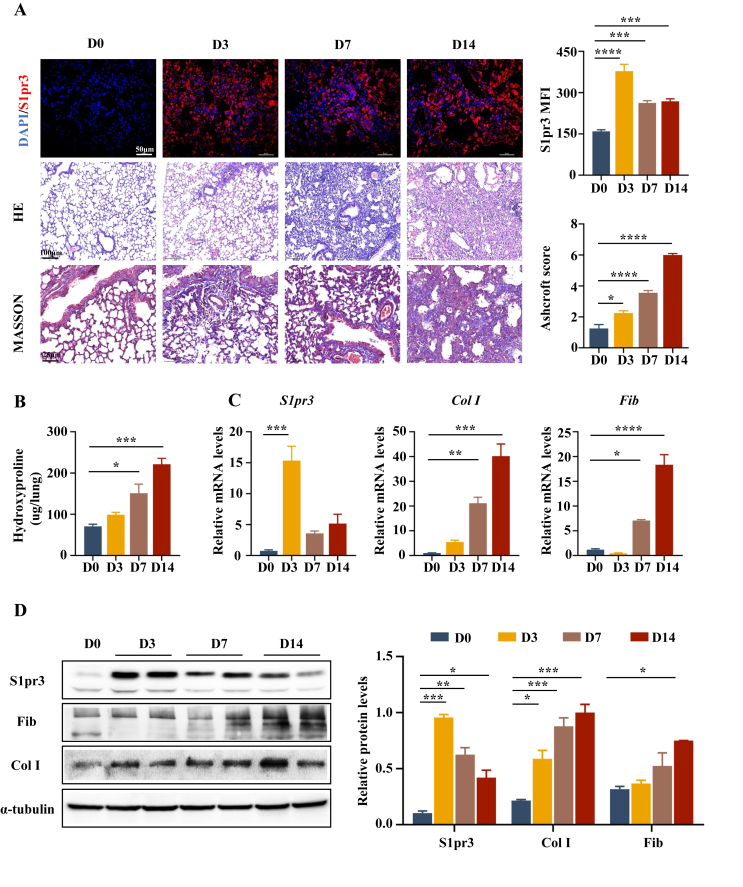
Analysis of S1pr3 expression in the development of BLM-induced pulmonary fibrosis in mice. **(A)** Representative images of S1pr3 immunofluorescence staining (upper panel, scale bar = 50 μm), HE staining (middle panel, scale bar = 100 μm), and Masson's trichrome staining (lower panel, scale bar = 25 μm) of lung tissues after BLM induction at different time points. The bar graph on the right shows quantitative S1pr3 expression and numerical fibrotic score (Ashcroft score). **(B)** Quantification of the hydroxyproline content of lung tissues at different time points after BLM administration. **(C, D)** Quantitative reverse transcription-PCR and Western blot analyses of S1pr3, fibronectin, and collagen I of lung homogenates. BLM, bleomycin; D0, normal control; D3/D7/D14, 3/7/14 days after BLM stimulation; MFI, mean fluorescence intensity; Col I, collagen I; Fib, fibronectin. ∗*p* < 0.05, ∗∗*p* < 0.01, ∗∗∗*p* < 0.001, ∗∗∗∗*p* < 0.0001.

We first established a PF model by intratracheal instillation of BLM and assessed the progression of fibrosis at different time points after BLM stimulation to examine the expression levels of S1pr3 in the development of PF. Immunofluorescence (Fig. 1A, top panel) showed that S1pr3 was almost undetectable in the lung sections from normal controls. BLM stimulation enhanced S1pr3 expression levels and showed the highest level at the early phase (3 days after BLM treatment) of the pathogenesis, along with progressive fibrotic changes displayed by HE- and Masson's trichrome-stained pathological slides (Fig. 1A, middle and bottom panels). Meanwhile, the Ashcroft scores and hydroxyproline levels increased over time (Fig. 1A, right panel, B). RT-PCR and Western blot data of lung homogenates also showed that S1pr3 expression levels were elevated during the development of fibrosis along with increased expression of collagen I (Col I) and fibronectin (Fib), two components of extracellular matrix,^25^ which are considered markers for fibrotic change (Fig. 1C, D). Together, these data suggest that S1pr3 expression is enhanced in the lungs of mice during the development of BLM-induced PF.

### S1pr3 expression is up-regulated in M2 macrophages of the lung during the development of BLM-induced PF in mice

**Figure 2:**
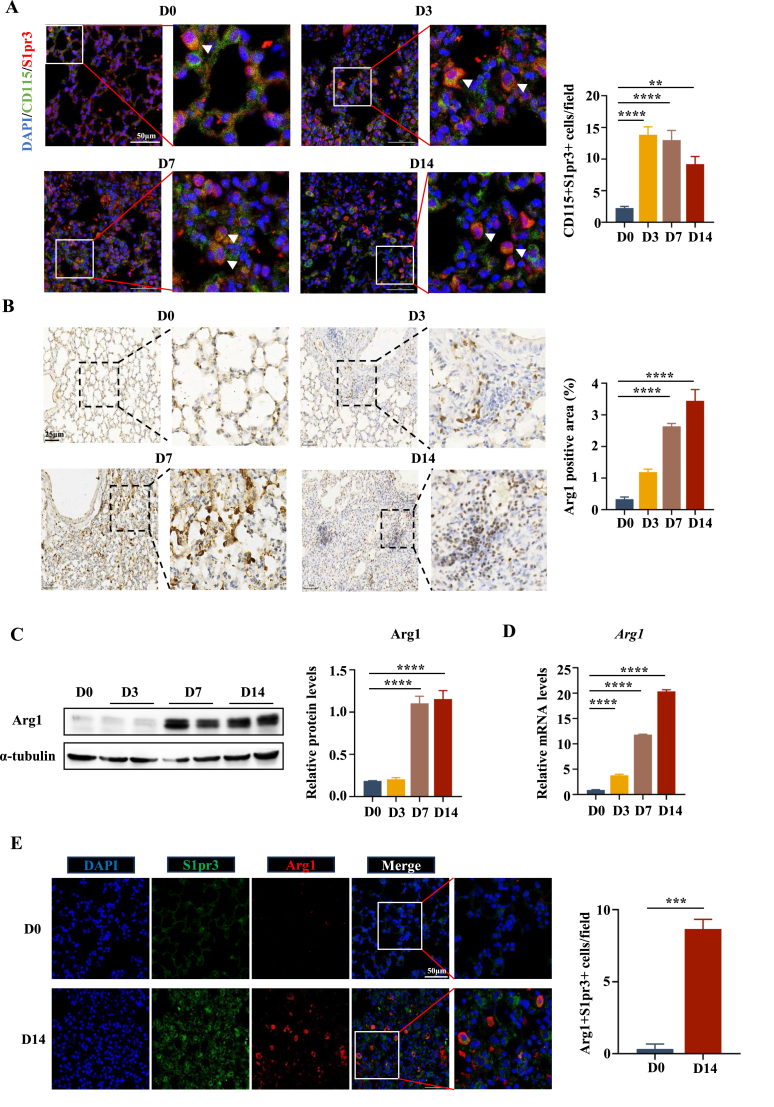
S1pr3 was overexpressed in macrophages and increased M2 macrophages in the lung following BLM induction. **(A)** Representative images of CD115 and S1pr3 co-immunostaining in BLM-induced lung sections at different time points (left panel, scale bar = 50 μm). Nuclei were stained with DAPI (blue). White arrows indicate CD115 and S1pr3 double-positive cells. **(B)** Representative images and quantitative analyses of IHC Arg1. Scale bar = 25 μm. **(C, D)** Western blot and quantitative reverse transcription-PCR analysis of Arg1 in lung homogenates. **(E)** Representative images of co-immunostaining of Arg1 and S1pr3 in normal lung sections (upper panel) and those 14 days after BLM induction (lower panel). Scale bar = 50 μm. IHC, immunohistochemistry; Arg1, arginase 1; DAPI, 4′,6-diamidino-2-phenylindole. ∗∗*p* < 0.01, ∗∗∗*p* < 0.001, ∗∗∗∗*p* < 0.0001.

Given the important role of macrophages in PF, immunofluorescence staining was performed to determine the cellular location of S1pr3 in the fibrotic lungs. S1pr3 expression levels were markedly increased in macrophages in mouse PF tissue compared with those in normal controls, marked by an increase in CD115 and S1pr3 double-positive cells in fibrotic lungs. In addition, the number of S1pr3-positive macrophages peaked on day 3 (Fig. 2A). Because M2 macrophages were highly correlated with PF development because of their pro-fibrotic effects, we next checked M2 macrophages in the lungs during BLM-induced PF. We performed immunohistochemical staining of arginase-1 (Arg1), as the marker of M2 macrophages.^26^^,^^27^ The results showed that Arg1-positive cells were rarely detected in normal control lungs and were abundantly increased in fibrotic lungs on days 7 and 14 (Fig. 2B). The same results were observed by Western blot analysis (Fig. 2C), while the mRNA levels of Arg1 started to increase on day 3, with a significant increase on days 7 and 14 (Fig. 2D). Next, to determine whether S1pr3 was involved in the M2 macrophage polarization in the fibrotic lungs, we performed co-immunostaining of S1pr3 and Arg1 in lung slides. Increased co-immunofluorescence of S1pr3 and Arg1 was detected on day 14 after BLM stimulation compared with almost undetectable fluorescence in normal controls (Fig. 2E), while the expression of S1pr3 was rare in M1 macrophages on day 14 after BLM stimulation revealed by the absence of co-immunofluorescence of S1pr3 and macrophage M1 marker iNOS (Fig. S1). Taken together, these results suggest that S1pr3 overexpression in M2 macrophages is associated with BLM-induced PF.

### Knockout of *S1pr3* in macrophages mitigates BLM-induced lung injury and fibrosis in mice

**Figure 3:**
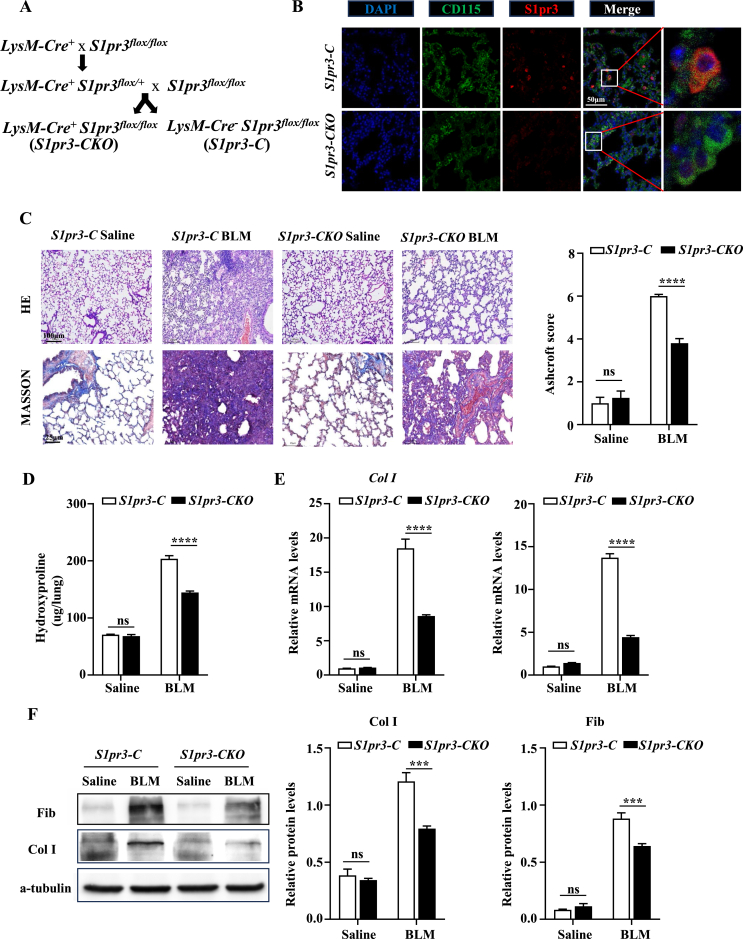
Conditional deletion of *S1pr3* in macrophages inhibited BLM-induced pulmonary fibrosis in mice. **(A)** Breeding strategy of *LysM-Cre*^*+*^*/S1pr3*^*flox/flox*^ (*S1pr3-CKO*) and *LysM-Cre*^*−*^*/S1pr3*^*flox/flox*^ (*S1pr3-C*) mice. **(B)** Representative images for co-immunostaining of CD115 and S1pr3 in lung sections from *S1pr3-C* and *S1pr3-CKO* mice on day 14 after BLM stimulation. Nuclei were stained with DAPI (blue); scale bar = 50 μm. **(C)** Representative images of HE staining (upper panel, scale bar = 100 μm) and Masson's trichrome staining (lower panel, scale bar = 25 μm) of lung tissues of *S1pr3-C* and *S1pr3-CKO* mice after BLM challenge. The bar graph shows the Ashcroft score. **(D)** Quantification of hydroxyproline content. **(E, F)** Quantitative reverse transcription-PCR and Western blot analysis of lung collagen I and fibronectin in *S1pr3-C* and *S1pr3-CKO* mice after BLM challenge. Col I, collagen I; Fib, fibronectin; DAPI, 4′,6-diamidino-2-phenylindole; ns, not significant. ∗∗∗*p* < 0.001, ∗∗∗∗*p* < 0.0001.

To investigate the role of S1pr3 in macrophages in the pathogenesis of PF, a myeloid-specific *S1pr3* knockout mouse model (the *LysM-Cre*^*+*^*/S1pr3*^*flox/flox*^ mice, defined as *S1pr3-CKO* mice) was generated, and their littermates (the *LysM-Cre*^*−*^*/S1pr3*^*flox/flox*^ mice, defined as *S1pr3-C* mice) served as controls (Fig. 3A). *S1pr3* depletion was confirmed by genotyping of the toe tissue DNA for the presence of the floxed allele (Fig. S2A) along with the detection of the Cre allele (Fig. S2B). S1pr3 deficiency in macrophages of *S1pr3-CKO* mice was validated by the absence of S1pr3 co-localization with CD115 positive macrophages, which was observed in the lung of *S1pr3-C* mice on day 14 after BLM stimulation (Fig. 3B). Since *LysM-Cre* is a myeloid-Cre line which can mediate deletion of genes in not only macrophages but also neutrophils,^28^ co-immunostaining of S1pr3 and Ly-6G was performed to check whether neutrophil S1pr3 was involved in the development of PF in mice. The results showed that S1pr3 also expressed on neutrophils while S1pr3 positive neutrophils were not significantly different at different time points after BLM challenge (Fig. S3), suggesting that neutrophil S1pr3 may not be involved in the development of BLM-induced PF. The *S1pr3-CKO* and *S1pr3-C* mice were used to assess the severity of PF 14 days after BLM stimulation. Significantly attenuated lung injury and fibrosis were noted in *S1pr3-CKO* mice, as demonstrated by HE and Masson's trichrome staining with lower Ashcroft scores (Fig. 3C). Phenotypically, the severity of PF was significantly lower in *S1pr3-CKO* mice than in *S1pr3-C* mice, as illustrated by the reduced hydroxyproline levels (Fig. 3D). In line with these observations, RT-PCR analysis confirmed a significant reduction in the expression of Fib and Col I in the lungs derived from *S1pr3-CKO* mice (Fig. 3E), and similar data were also obtained by Western blot (Fig. 3F). The results also showed no significant difference in lung phenotype between *S1pr3-CKO* and *S1pr3-C* mice under the basic state. Overall, these data support the hypothesis that specific deletion of *S1pr3* in macrophages ameliorates BLM-induced lung damage and fibrosis in mice.

### *S1pr3* deficiency attenuates macrophage M2 polarization

**Figure 4:**
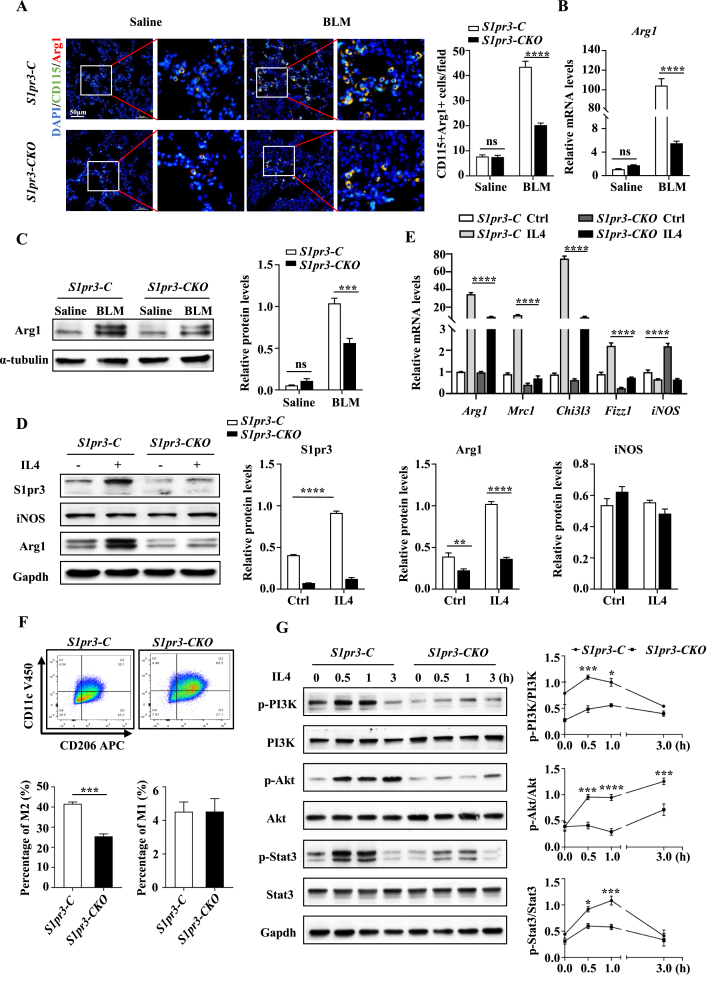
Loss of *S1pr3* inhibited macrophage M2 polarization. **(A)** Representative images for co-immunostaining of CD115 and Arg1 in lung sections of *S1pr3-C* and *S1pr3-CKO* mice after BLM challenge, Scale bar = 50 μm. **(B, C)** Quantitative reverse transcription-PCR and Western blot analysis of lung Arg1 in *S1pr3-C* and *S1pr3-CKO* mice. **(D)** Western blot results for S1pr3, iNOS, and Arg1 expression in BMDMs after IL-4 induction. **(E)** Quantitative reverse transcription-PCR analysis of *Arg1*, *Mrc1*, *Chi3l3*, *Fizz 1*, and *iNOS* expression in BMDMs after IL-4 induction. **(F)** Flow cytometry analysis of the percentage of M1 macrophages (CD11b^+^F4/80^+^CD11c^+^CD206^−^ cells) and M2 macrophages (CD11b^+^F4/80^+^CD11c^−^CD206^+^ cells) in BMDMs following IL-4 stimulation. **(G)** Time-course Western blot analysis of IL-4-induced PI3K/Akt-Stat 3 signaling activity in BMDMs. BMDMs, bone marrow-derived macrophages; Arg1, arginase 1; Mrc1, mannose receptor 1; Chi3l3, chitinase 3-like 3; Fizz1, found in inflammatory zone 1; ns, not significant. ∗*p* < 0.05, ∗∗*p* < 0.01, ∗∗∗*p* < 0.001, ∗∗∗∗*p* < 0.0001.

We next investigated whether conditional loss of *S1pr3* in myeloid cells could influence M2 macrophage infiltration in fibrotic lungs. Co-immunostaining of Arg1 and CD115 revealed that M2 macrophages were significantly reduced in lung tissues of *S1pr3-CKO* mice (Fig. 4A). The same result was also found by the immunohistochemical staining of Arg1 in lung sections of *S1pr3-C* and *S1pr3-CKO* mice (Fig. S4). Moreover, RT-PCR analysis showed decreased expression of *Arg1* in *S1pr3-CKO* mice compared with *S1pr3-C* mice (Fig. 4B). Consistently decreased Arg1 protein levels were observed in *S1pr3-CKO* mice by Western blot (Fig. 4C), which indicated that *S1pr3-CKO* mice had less M2 macrophage infiltration in the lung than *S1pr3-C* mice. Collectively, these findings demonstrate that *S1pr3* can affect the progression of PF by affecting the infiltration of M2 macrophages in the lung.

The above results prompted us to address the effect of *S1pr3* on macrophage M2 polarization. Primary bone marrow cells were isolated and cultured with supplementary macrophage colony-stimulating factor to differentiate into macrophages (Fig. S5A, B), and the purity of BMDMs was detected by immunofluorescence staining of F4/80 (Fig. S5C). We first tested the effect of IL-4 stimulation on the mRNA expression of S1P receptors in BMDMs. The results showed that IL-4 stimulation induced the mRNA expression of S1pr3 but not S1pr1 and S1pr2 (Fig. S6A). Western blot analysis also demonstrated elevated S1pr3 expression after IL-4 stimulation (Fig. S6B). RT-PCR and Western blot analysis verified that IL-4 polarized macrophages to the M2 phenotype, as illustrated by the increase of the M2 marker Arg1 expression, while it had no significant effect on the macrophage M1 polarization, as illustrated by no alteration of the M1 marker iNOS (Fig. S7). Because S1pr3 is the receptor for S1P, we next attempted to determine the effect of additional S1P supplementation on macrophage polarization. We found that S1P did not significantly alter the expression of macrophage polarization markers or affect IL-4-induced macrophage M2 polarization (Fig. S7). In other words, additional S1P supplementation had no significant effect on macrophage polarization. Therefore, in the following experiments, IL-4 was used alone to induce macrophage M2 polarization. Consistent with the results in BMDMs, IL-4 stimulation also enhanced the expression of S1PR3 in THP-1 (human monocyte cell line) derived macrophages along with increased expression of M2 markers (*MRC1*, *ALOX15*, *CCL17*, and *CD200R)* (Fig. S8), indicating that S1PR3 may also be involved in human macrophage M2 polarization.

To further dissect the role that *S1pr3* played in the regulation of macrophage M2 polarization, BMDMs were generated from *S1pr3-C* and *S1pr3-CKO* mice and then subjected to IL-4 stimulation. Remarkably, IL-4 induced significantly higher levels of Arg1 in *S1pr3-C* BMDMs than in *S1pr3-CKO* BMDMs, and a substantial increase in S1pr3 protein level was noted upon IL-4 stimulation in *S1pr3-C* BMDMs. Additionally, there was no significant difference in iNOS protein levels between *S1pr3-C* and *S1pr3-CKO* BMDMs (Fig. 4D). RT-PCR analysis also revealed lower expression of M2 markers (*Arg1*, *Mrc1*, *Chi3l3*, and *Fizz1*) in *S1pr3-CKO* BMDMs than in *S1pr3-C* BMDMs following IL-4 induction (Fig. 4E). Interestingly, higher iNOS mRNA levels and lower Arg1 protein levels were noted in *S1pr3-CKO* BMDMs than in *S1pr3-C* BMDMs under basic conditions, suggesting that S1pr3 deficiency alone may predispose macrophages to the M1 phenotype. BMDMs were also subjected to flow cytometry analysis following IL-4 stimulation. Similar to the results of RT-PCR and Western blot, *S1pr3-CKO* BMDMs displayed a significantly lower percentage of M2 macrophages (CD206^+^CD11c^−^) than *S1pr3-C* BMDMs, while there was no significant difference in the proportion of M1 macrophages (CD206^−^CD11c^+^) (Fig. 4F). Taken together, these findings indicate that *S1pr3* deficiency can attenuate IL-4-induced macrophage M2 polarization in BMDMs.

### Deletion of *S1pr3* represses the PI3K/Akt-Stat3 signaling pathway to attenuate the M2 polarization

STAT3 activation is critical for macrophage M2 polarization, dependent on PI3K/AKT pathway activation,^29^ and S1pr3 has been reported to influence both of these signals under a variety of physiological and pathological states. Therefore, we next compared the temporal expression changes in terms of the PI3K/Akt-Stat3 signaling pathway between *S1pr3-CKO* and *S1pr3-C* BMDMs following IL-4 stimulation to determine the mechanism of *S1pr3* regulation of macrophage M2 polarization. IL-4 stimulation promoted the time-dependent increase in p-PI3K and p-Akt in BMDMs, and the degree of promotion in *S1pr3-C* BMDMs was significantly higher than that in *S1pr3-CKO* BMDMs. Stat3 phosphorylation levels were increased in the first hour of IL-4 stimulation and decreased to basic levels at 3 h following IL-4 induction. Moreover, higher p-Stat3 levels were observed in *S1pr3-C* BMDMs than in *S1pr3-CKO* BMDMs (Fig. 4G). These results indicate that *S1pr3* affects macrophage M2 polarization by regulating the PI3K/Akt-Stat3 signaling pathway.

### S1pr3-specific inhibitors protect mice against BLM-induced PF

**Figure 5:**
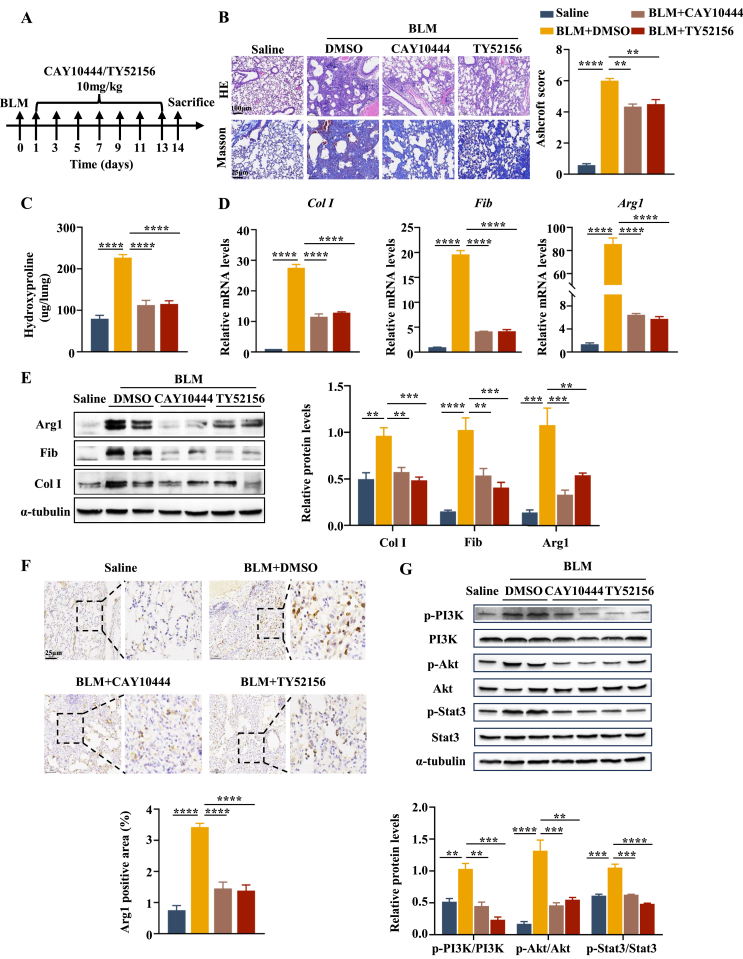
S1pr3 inhibitors protect mice from BLM-induced lung injury and fibrosis. **(A)** Schematic of the experimental protocol for bleomycin exposure, S1pr3 inhibitor treatment, and endpoints in mice with bleomycin-induced lung fibrosis; 2 mg/kg BLM was administered intratracheally to induce pulmonary fibrosis. On the day after bleomycin injection, the S1pr3 inhibitor CAY10444 or TY52156 at 10 mg/kg was intraperitoneally administered every other day for seven times, and mice were sacrificed on day 14. **(B)** Representative images of HE staining (upper panel, scale bar = 100 μm) and Masson's trichrome staining (lower panel, scale bar = 25 μm) of lung tissues in different treatment groups. The bar graph shows the Ashcroft score. **(C)** Quantification of hydroxyproline content. **(D, E)** Quantitative reverse transcription-PCR and Western blot analysis of lung fibronectin, collagen I, and Arg1. **(F)** Representative images and quantitative analysis of IHC Arg1 in different treatment groups. Scale bar = 25 μm. **(G)** Western blot analysis of PI3K/Akt-Stat3 signaling in lung tissues of different groups. BLM, bleomycin; Col I, collagen I; Fib, fibronectin; Arg1, arginase1. ∗∗*p* < 0.01, ∗∗∗*p* < 0.001, ∗∗∗∗*p* < 0.0001.

Finally, the therapeutic potentials of the two S1pr3-specific inhibitors CAY10444 and TY52156 were tested. Because S1pr3 was observed to peak on day 3 after BLM stimulation, CAY10444 or TY52156 was intraperitoneally administered on day 1 after BLM challenge and was administered every other day until sacrifice on day 14 (Fig. 5A). Administration of CAY10444 or TY52156 both alleviated BLM-induced PF significantly, as illustrated by histopathological analysis and Ashcroft scores (Fig. 5B). Consistently, lower levels of hydroxyproline were observed in the lungs of mice treated with S1pr3 inhibitors (Fig. 5C), which was coupled with a significant reduction in fibrotic markers (Fib and Col I) and macrophage M2 marker (Arg1) at both the mRNA and protein levels (Fig. 5D, E). Immunohistochemical staining of Arg1 also showed less M2 macrophage infiltration in the lung after treatment with S1pr3 inhibitors (Fig. 5F). We next assessed whether S1pr3 inhibitors regulate the polarization of M2 macrophages via PI3K/Akt-Stat3 signaling pathway *in vivo* consistent with the results obtained in BMDMs *ex vivo*. Western blot analysis showed that S1pr3 inhibitors can attenuate BLM-induced activation of the PI3K/Akt-Stat3 signaling pathway in lung tissues (Fig. 5G). Taken together, these results illustrated that the administration of S1pr3 inhibitors in the early inflammatory phase of BLM-induced lung injury would ameliorate the formation of fibrosis by reducing the polarization of macrophage into M2 phenotype, suggesting that S1pr3-specific inhibitors represent a viable intervention approach against PF.

## Discussion

In the current research, we conducted experiments to explore the effect of S1pr3, a receptor for the lipid signaling molecular S1P, on the pathogenesis of PF. S1pr3 was increased in whole lung tissues and specifically overexpressed in the pulmonary infiltrated M2 macrophages of BLM-induced PF in mice. Compared with wild-type mice, *S1pr3-CKO* mice had less M2 macrophage accumulation in the lungs, and the degree of PF and lung damage was less severe after BLM challenge. *Ex vivo* experiments also demonstrated that deletion of *S1pr3* attenuated IL-4-induced macrophage M2 polarization, which may be related to the repression of PI3K/Akt-Stat3 activity. Finally, the S1pr3 inhibitors CAY10444 and TY52156 exerted protective effects on PF in mice after BLM challenge.

As previously reported, the S1P-S1PR signaling pathway is highly involved in the pathogenesis of fibrosis. S1P levels have been shown to be elevated in serum and bronchoalveolar lavage fluid in patients with idiopathic PF compared with control groups, and high S1P levels in bronchoalveolar lavage fluid are correlated with poor pulmonary function. S1P is highly profibrotic and manifests by inducing epithelial to mesenchymal transition, causing myofibroblast differentiation, and promoting excessive production of extracellular matrix by activating S1pr2 and S1pr3.^18^^,^^30^^,^^31^ Controlling S1P levels or deleting S1pr2/S1pr3 can reduce PF, irrespective of whether it is BLM-induced or radiation-induced.^19^^,^^20^^,^^30^^,^^32^, ^33^, ^34^ However, none of these studies involved changes in the expression levels of S1pr3 at different stages of fibrosis formation. In our study, the expression of S1pr3 in the lungs was elevated during the entire process of PF and peaked at the inflammatory stage, approximately 3 days after BLM instillation, which suggested that S1pr3 may contribute to PF development through interacting with inflammatory cells.

According to origins, lung macrophages are classified as tissue-resident or monocyte-derived.^11^ Tissue-resident macrophages undergo self-renewal in the steady state without contribution from circulating monocytes.^35^, ^36^, ^37^ During injury, monocytes are recruited into the lung and differentiate into monocyte-derived macrophages.^27^^,^^38^ In PF models, monocyte-derived macrophages instead of tissue-resident macrophages are necessary for developing fibrosis.^39^ Monocyte-derived macrophages are polarized to the alternatively activated (M2) phenotype to promote the fibrotic process by releasing cytokines and multiple growth factors.^40^ M2 macrophage infiltration is a common characteristic of PF, as shown in both PF patients and PF mouse models.^12^^,^^40^^,^^41^ Consistent with previous research, increased M2 macrophage infiltration into the lungs was also found in our study during the development of BLM-induced PF in mice. Thus, inhibiting macrophage polarization to the M2 phenotype is a viable treatment option for PF. The S1P system is an important regulator of macrophage function. The effect of S1P on macrophages is complex because of the engagement of different receptors in different situations. Among all five receptors, our research focused on S1pr3 since S1pr3 expression is both high and specific in monocytes and plays a role in myeloid differentiation.^20^^,^^42^ S1pr3 has been shown to be increased during monocyte differentiation to macrophages, and S1pr3^hi^ monocytes are progenitors of wound healing M2 macrophages in skin.^43^^,^^44^ Monocyte-derived macrophages peaked at day 3 after BLM challenge^45^ and we found that S1pr3 expression also peaked at day 3 after BLM challenge (Fig. 1), suggesting that S1pr3 contributes to fibrosis development by affecting monocyte-derived macrophages. Therefore, we conducted co-immunostaining of S1pr3 and CD115 (macrophage marker) and confirmed the elevation of S1pr3 expression in macrophages after BLM challenge. Due to the pro-fibrotic effect of M2 macrophages on PF, we also checked S1pr3 expression in M2 macrophages (marked by Arg1) and found that S1pr3-positive M2 macrophages were significantly increased in fibrotic mouse lungs compared with normal controls (Fig. 2). Inhibition of S1pr3 through knockout or inhibitors both attenuated lung injury and fibrosis combined with decreased M2 macrophage infiltration (Figure 3, Figure 5). *LysM-Cre* as a myeloid-Cre line was employed in our study to induce a selective deletion in macrophages. Although S1pr3 positive neutrophils were found to be unchangeable during the development of BLM-induced PF (Fig. S3), future studies employing more macrophage-specific Cre lines such as *Cx3cr1-Cre* are still necessary to confirm our results obtained from the *LysM-Cre* system. S1pr3 inhibitors were used at the early inflammatory phase of BLM challenge to treat the mice, indicating that these can exert the potent anti-inflammation effect to attenuate the formation of fibrosis. Further studies are required to determine whether S1pr3 inhibitors can reverse the established fibrosis.

IL-4 is a recognized inducer of macrophage M2 polarization. In our study, primary BMDMs were used *ex vivo* to further dissect the role of S1PR3 in macrophage polarization. Consistent with a previous report,^21^ we found that IL-4 stimulation increased S1pr3 expression and induced M2 phenotype polarization of macrophages (Fig. 4D; Fig. S6), implying that S1pr3 is involved in the regulation of macrophage M2 polarization. Intriguingly, additional S1P supplementation seemed to not affect the polarization of macrophages *ex vivo*, as evidenced by similar expression levels of Arg1 and iNOS following treatment with S1P alone or S1P combined with IL-4 (Fig. S7). This is consistent with the findings of Müller et al,^46^ which demonstrated that S1P did not significantly alter the percentage of M2 macrophages under either M1- or M2-polarizing conditions. However, another study mentioned that S1P alone could polarize macrophages to the M1 phenotype.^47^ This discrepancy may arise from the species variation, which means that the BMDMs from different kinds of mice with various gene expression profiles, such as C57BL/6 and ICR, may have distinct responses to the stimulation of S1P. In addition, macrophages can produce S1P by themselves through the mediation of sphingosine kinases.^48^ Hence, we speculate that S1P is sufficient in the *ex vivo* culture system of BMDMs, resulting in the unresponsiveness of macrophages to S1P supplementation. Further studies are needed to confirm this speculation. Overall, S1P did not significantly affect macrophage polarization under the basic state or during IL-4 stimulation in our study. With regard to BMDMs from *S1pr3-CKO* or *S1pr3-C* mice, we found that loss of *S1pr3* attenuated IL-4-induced macrophage M2 polarization. Some other studies have shown that the activation of S1pr3 promoted the M1 polarization of macrophages.^49^^,^^50^ Because macrophages are highly plastic, differences in sources, gene expression profiles, and *in vitro* culture protocols may lead to contradictory roles of S1pr3 in the polarization of macrophages among studies. Indeed, mouse peritoneal macrophages and primary microglia were used in these two experiments, and the methods used to silence S1pr3 were also different from those in the current study. Interestingly, we found that the mRNA level of iNOS was increased in *S1pr3-CKO* BMDMs, which was not reflected in the protein level. This contradictory observation cannot be clarified at present, but it has been suggested that the association between protein and mRNA expression levels was quite low in mammals.^51^ Post-transcriptional regulation, delayed synthesis between mRNA and protein, and measurement noise may cause this weak correlation,^52^ and particularly, iNOS is regulated transcriptionally and post-transcriptionally by an intricate array of transcription factors, non-coding RNAs, and post-translational modification.^53^

S1PR3 regulates multiple physiological processes through both the PI3K/AKT and STAT3 signaling pathways. S1PR3 can regulate the proliferation of endothelial progenitor cells and the vasodilation effect by activating the PI3K/AKT pathway.^54^^,^^55^ S1PR3 also plays a protective role in atherosclerosis by promoting endothelial cell migration and inhibiting the apoptosis of macrophages through PI3K/AKT and STAT3/Survivin signals, respectively.^56^^,^^57^ The roles of PI3K/AKT in the activation of macrophages have been mentioned in many articles. AKT appears to promote M2 polarization because the expression of M2-related genes is inhibited by pharmacological inhibition of AKT.^58^ Moreover, increased macrophage M2 polarization in emphysematous mice was found to be mediated by activation of the PI3K/AKT pathway.^59^ STAT3 is also known to play a critical role in the M2 phenotype polarization of macrophages. Elimination of STAT3 inhibits the polarization of human monocyte-derived macrophages to the M2 phenotype, whereas activation of STAT3 promotes the M2 polarization of macrophages in atopic dermatitis and PF mouse models.^41^^,^^60^ Furthermore, some studies have shown that STAT3 activation relies on the activation of the PI3K/AKT pathway in macrophages. In addition, the activation of PI3K/AKT-STAT3 facilitates the polarization of macrophages toward M2, exerting immunomodulatory effects.^29^^,^^61^^,^^62^ Therefore, we evaluated PI3K/Akt-Stat3 activation states to determine whether S1pr3 regulates macrophage M2 polarization via this signaling pathway. The data showed that the lack of *S1pr3* reduced IL-4-induced activation of the PI3K/Akt-Stat3 pathway, suggesting a regulative effect of S1pr3 on IL-4-induced macrophage M2 polarization through the PI3K/Akt-Stat3 signaling pathway.

Immunostaining of lung sections suggested that S1pr3 levels were also increased in alveolar type II epithelial cells and mesenchymal cells in BLM-induced PF mouse lungs (Fig. S9). Given that macrophages were the main cell type used in this study, the effect of alterations in S1pr3 expression in alveolar type II epithelial cells and mesenchymal cells in PF will be explored in our future study.

## Conclusions

S1pr3 contributes to PF by modulating macrophage M2 polarization through the PI3K/Akt-Stat3 signaling pathway. In addition, S1pr3 inhibitors were found to be effective for mouse PF, suggesting that these represent a potential treatment option for patients with PF.

## CRediT authorship contribution statement

Huijun Qiu designed and performed the experiments and wrote the manuscript. Jiang Liu, Jingyi You, Ou Zhou, Chang Hao, and Deyu Ma performed part of the experiments. Yi Shu contributed to study design and manuscript editing. Wenjing Zou, Linghuan Zhang, Zhengxiu Luo, Enmei Liu, Luo Ren, and Gang Geng revised the manuscript. Lin Zou, Zhou Fu, and Danyi Peng contributed to study design, financial support, data analysis and interpretation, editing and revising of the manuscript, and final approval.

## Conflict of interests

The authors declared that they had no competing interests.

## Funding

This work was supported by the General Basic Research Project from the Ministry of Education Key Laboratory of Child Development and Disorders (China) (No. GBRP-202115) and the Chongqing Science and Technology Bureau Major Project (China) (No. cstc2020jcyj-msxmX0782).
