# Supplementary material for: Inhibition of sphingosine 1-phosphate receptor 3 ameliorates bleomycin-induced pulmonary fibrosis by suppressing macrophage M2 polarization
Source: Genes Dis. 2024 Feb 15;12(3):101244. doi: 10.1016/j.gendis.2024.101244 (PMC11907442; doi:10.1016/j.gendis.2024.101244)
Supplement: Multimedia component 1 [file mmc1.pdf]

Fig S1

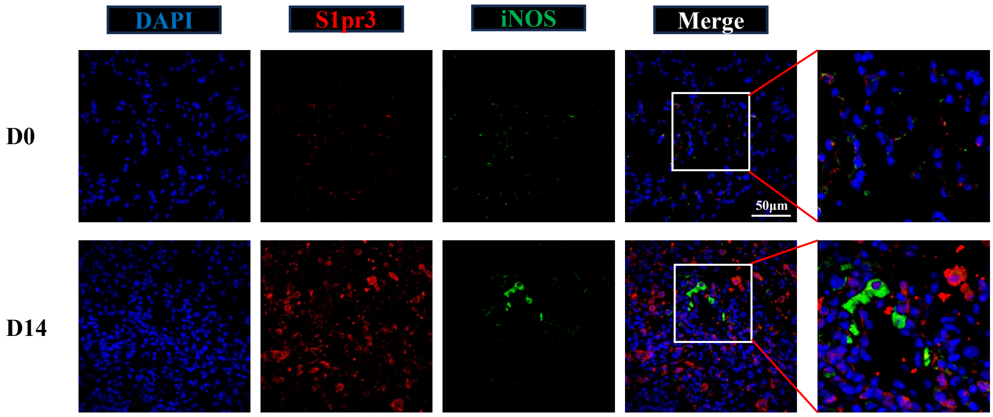

Fig. S1. Co-immunostaining of S1pr3 with M1 macrophages. Representative images of co-immunostaining of iNOS and S1pr3 in normal lung sections (upper panel) and those 14 days after BLM induction (lower panel). Scale bar = 50 μm.

Fig S2

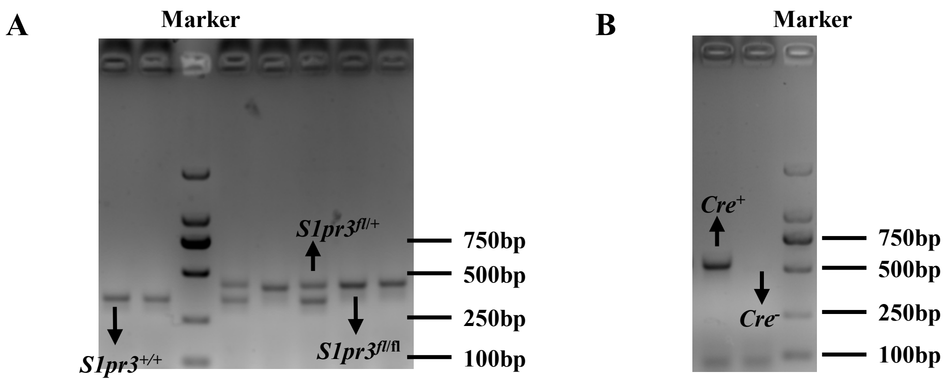

Fig. S2. Genotyping results of the *S1pr3-flox* and *LysM-Cre* alleles of mice. (A) The *S1pr3-flox* allele is indicated by arrows. (B) The band for the *LysM-Cre* allele.

Fig S3

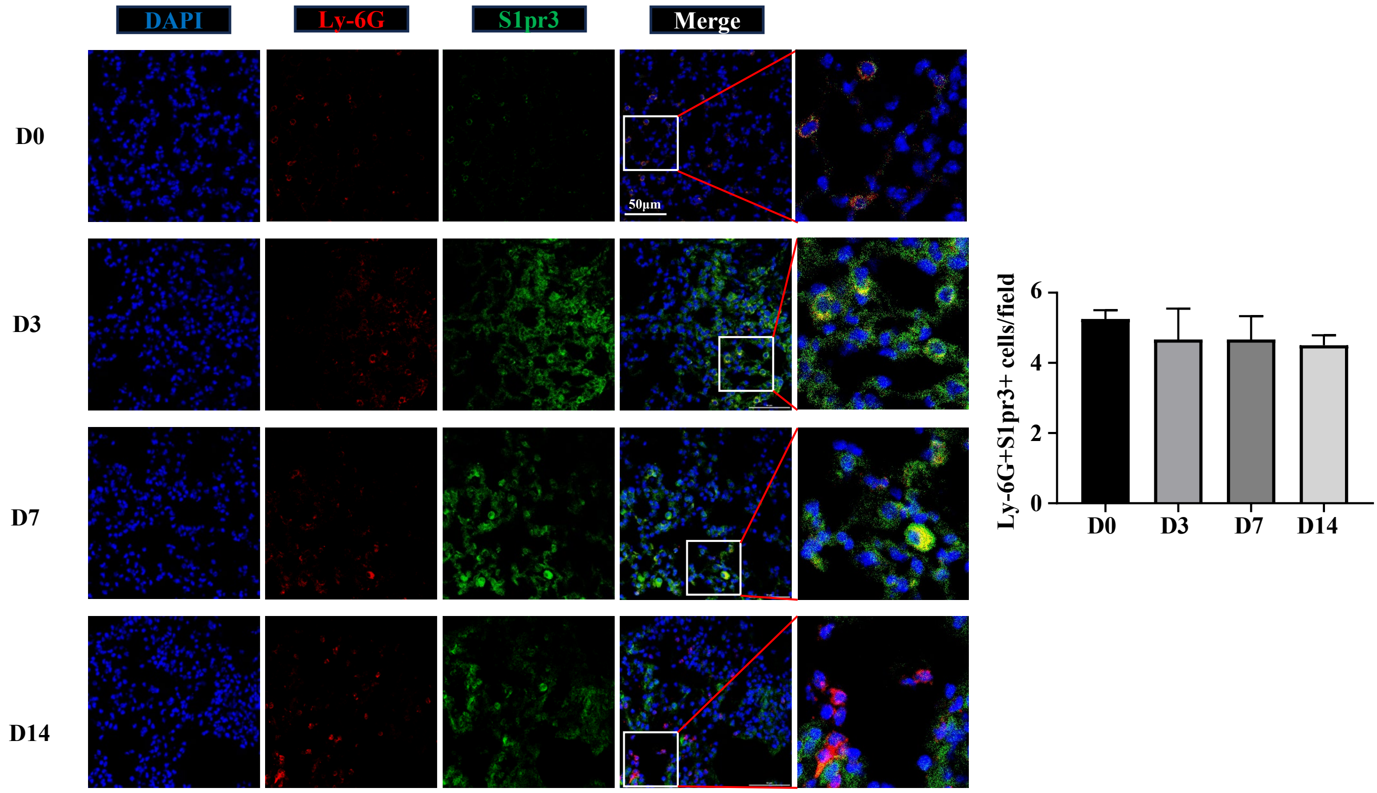

Fig. S3. Co-immunostaining of S1pr3 with neutrophils. Representative images of S1pr3 co-immunostaining with Ly-6G positive neutrophils in BLM-induced lung sections at different time points. Scale bar = 50 µm.

Fig S4

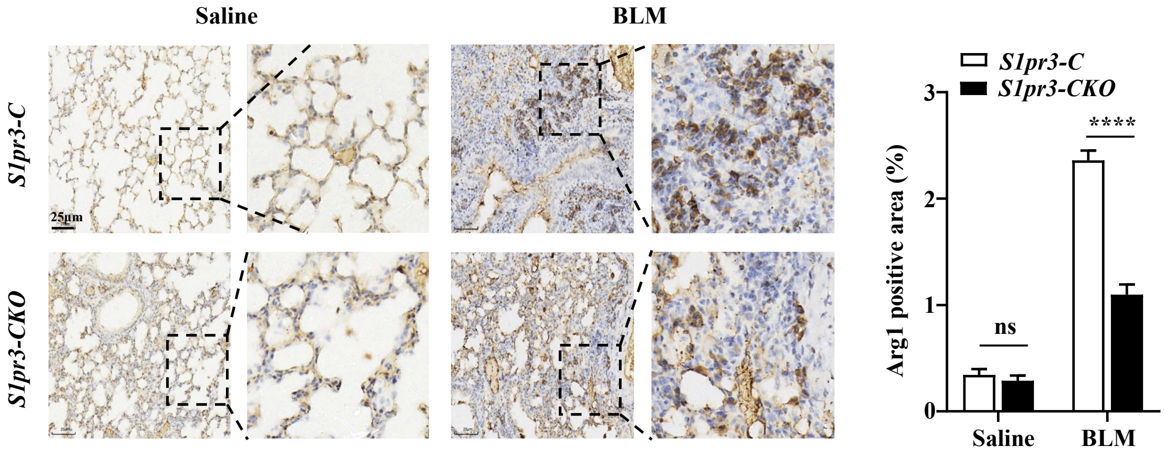

Fig. S4. Immunohistochemistry analysis of lung Arg1. (A) Representative images and (B) statistical analysis of IHC staining of lung Arg1 in *S1pr3-C* and *S1pr3-CKO* mice after BLM challenge. Scale bar = 25 μm. Arg 1: Arginase 1, ns: not significant; ****P < 0.0001.

Fig S5

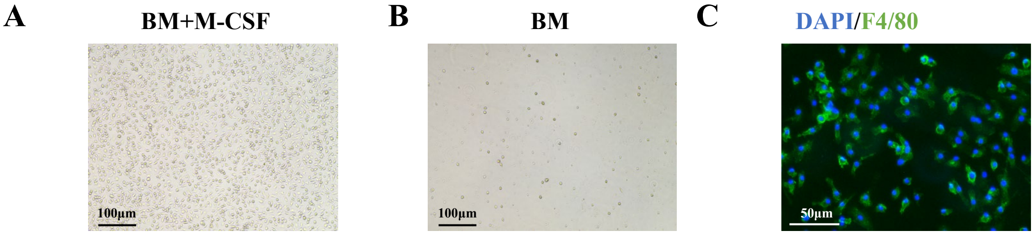

Fig. S5. Morphology and F4/80 staining of BMDMs. (A–B) Morphology of primary BM cells cultured with (A) or without (B) M-CSF for 7 days. scale bar = 100 μm. (C) Representative immunofluorescence images of primary BMDMs stained with FITC-labeled F4/80, scale bar = 50 μm. BMDMs: Bone marrow-derived macrophages.

Fig S6

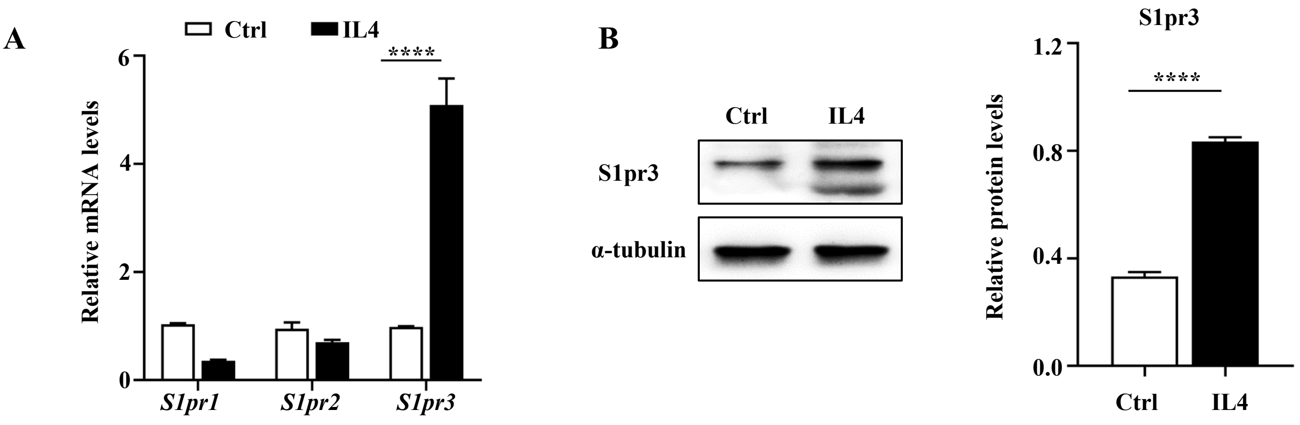

Fig. S6. Increased S1pr3 expression in IL4-induced BMDMs. (A) RT-PCR analysis of *S1pr1*, *S1pr2*, and *S1pr3* in BMDMs after IL-4 (20 ng/mL) stimulation for 24 h. (B) Western blot analysis of S1pr3 in BMDMs after IL-4 (20 ng/mL) stimulation for 24 h. BMDMs: Bone marrow-derived macrophages; ****$P < 0.0001$.

Fig S7

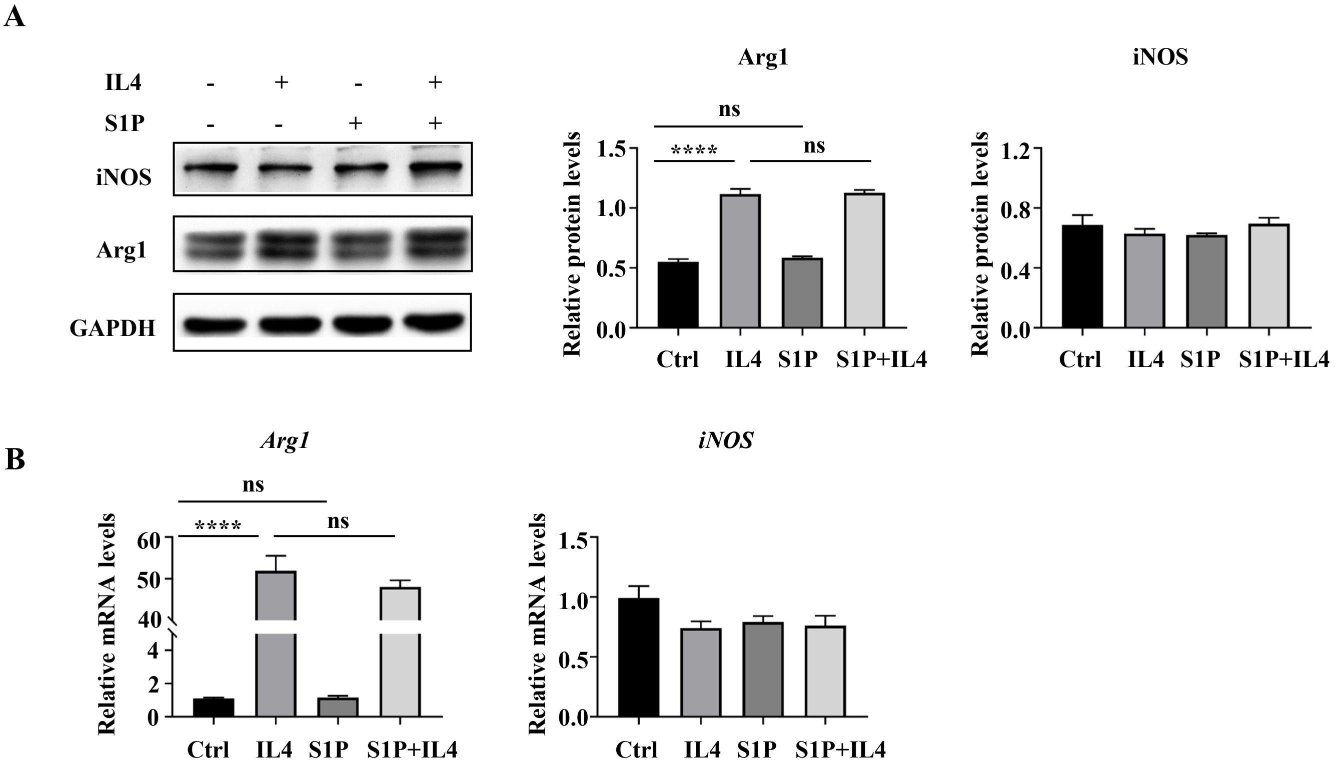

Fig. S7. Effect of IL-4 and S1P on macrophage polarization in BMDMs. Western blot (A) and RT-PCR (B) analysis of iNOS and Arg1 expression in BMDMs after different stimulation treatments for 24 h. BMDMs: Bone marrow-derived macrophages, Arg 1: Arginase 1, ns: not significant; ****P < 0.0001.

Fig S8

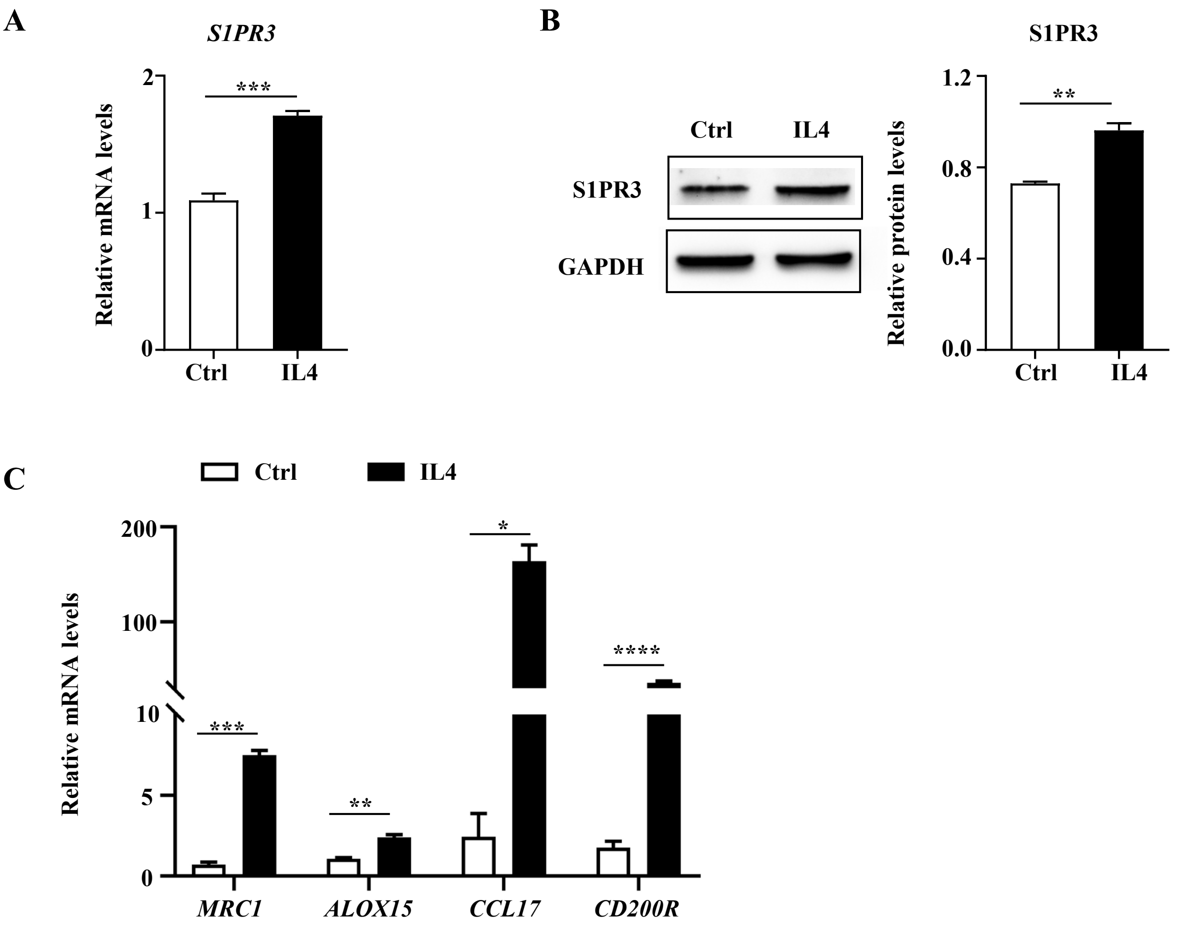

Fig. S8. Increased S1pr3 expression in IL4-induced THP-1 cells. (A-B) RT-PCR and Western blot analysis of S1PR3 in THP-1 derived macrophages after IL-4 (20 ng/mL) stimulation. (C) RT-PCR analysis of human M2 macrophage markers in THP-1 derived macrophages after IL-4 (20 ng/mL) stimulation. *$P < 0.05$, **$P < 0.01$, ***$P < 0.001$, and ****$P < 0.0001$.

Fig S9

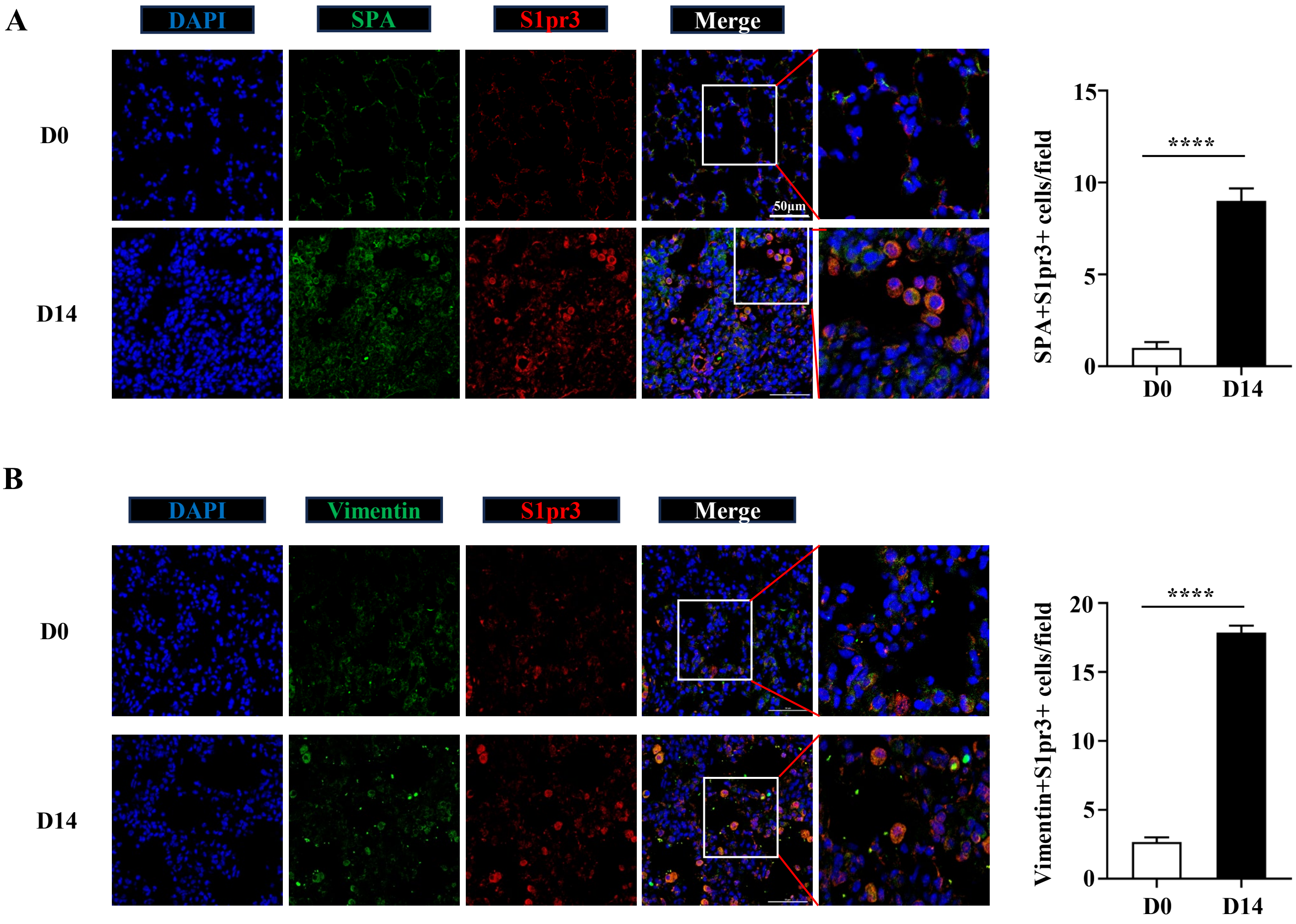

Fig. S9. Co-immunostaining of S1pr3 with AT II and mesenchymal cells. Representative images of S1pr3 co-immunostaining with AT II cells (A) and mesenchymal cells (B) in lung sections, Scale bar = 50 μm. ****$P < 0.0001$..
